# Long-Term Hepatitis B Vaccine Immunity in Ethiopian Children That Received a Pentavalent Vaccine Series: A Retrospective Cohort Study

**DOI:** 10.3390/children11010136

**Published:** 2024-01-22

**Authors:** Tinsae Alemayehu, Million Dechassa Daba, Danilo Buonsenso

**Affiliations:** 1 Department of Pediatrics and Adolescent Health, University of Botswana, Private Bag UB, Gaborone 00713, Botswana; alemayehut@ub.ac.bw; 2 Department of Pediatrics and Adolescent Health, St. Paul’s Hospital Millennium Medical College, Addis Ababa P.O. Box 1271, Ethiopia; million.dechasa@sphmmc.edu.et; 3 Department of Woman and Child Health and Public Health, Fondazione Policlinico Universitario A. Gemelli IRCCS, 00168 Rome, Italy; 4 Centro di Salute Globale, Università Cattolica del Sacro Cuore, 00168 Rome, Italy

**Keywords:** hepatitis B, Ethiopia, children, breakthrough infections

## Abstract

Background: Chronic hepatitis B affects close to 300 million people globally with 1.5 million new infections per year. It causes the highest numbers of cirrhosis and liver cancer diagnoses each year. In children, perinatal transmission and contact with infected blood or body fluids remain the main methods of transmission. There are increasing reports of breakthrough hepatitis B infections in fully vaccinated children born to hepatitis B-negative mothers, especially in low- and middle-income countries. Our study aimed to measure the adequacy of hepatitis B surface antibody levels among children and adolescents who received three rounds of hepatitis B vaccination during infancy and delivered to hepatitis B-negative mothers in Addis Ababa, Ethiopia. Method: This was a retrospective cohort study analyzing results of paired serology tests for hepatitis B surface antibody and antigen tests performed for children aged 1–18 years from July 2022 to June 2023. All recorded data were transferred to SPSS version 29.0. The prevalence of adequate hepatitis B surface antibody levels was determined and sub-group analysis conducted using descriptive statistics, frequencies and tables. The magnitude of association between different variables and vaccine-induced hepatitis B immunity was assessed using logistic regression. Statistically significant differences were taken at *p* < 0.05. Results: A total of 256 children were included in the study (mean age: 7.53 years). Six children (2.3%) had breakthrough hepatitis B infections. Overall, 37 children (14.4%) were categorized as having optimal hepatitis B surface antibody levels (vaccine-induced antibody titers of >10 IU/mL), while 219 (85.6%) had low titers of <10 IU/mL. Nearly all (97.4%) of the sub-group aged 10 years and above had below-par antibody levels, with adolescents (11–18 years) being ten times more likely to have low seroprotection than those aged less than 5 years. Conclusions: Our study showed markedly low vaccine-induced hepatitis B surface antibody levels among the study population, especially adolescents. The presence of breakthrough infections may suggest a genuine lack of response and not just a mere drop in antibody titers and thus could highlight a significant public health problem in Ethiopia. Further immunologic studies and a thorough analysis of vaccine storage and administration should be conducted to inform prevention programs.

## 1. Introduction

Chronic hepatitis B affects close to 300 million people globally (1.5 million new infections per year) and causes the highest numbers of cirrhosis and liver cancer diagnoses each year [1]. In children, perinatal transmission and contact with infected blood or body fluids remain the main methods of transmission. There are increasing reports of breakthrough hepatitis B infections in fully vaccinated children born to hepatitis B-negative mothers [2]. Childhood hepatitis B infections lead to cirrhosis (1% to 5% of cases) and hepatocellular carcinoma (2% to 5%) during childhood [2,3].

The national vaccination schedule in Ethiopia has included hepatitis B vaccines as part of a three-dose pentavalent vaccine series for infants since 2005, with the first dose administered at 45 days of life. Studies in the immediate pre-vaccine introduction years showed intermediate endemicity for hepatitis B infection with 5–6% of the population having been chronically infected [4]. A decade after introduction, community-based seroprevalence studies among Ethiopian school-age children (6–10 years) in urban areas showed prevalence rates of hepatitis B infection based on serum hepatitis B surface antigen (HBsAg ELISA) of 0.4–4.6% [5,6,7,8,9]. A concerning note these studies strike is the early loss of vaccine immunity in the study population’s age range. Alarming drops in vaccine immunity were seen with 42–80% of the studied children having non-protective anti-HBs titers of <10 IU/mL. Reflective of this was the finding that 1–2% of fully vaccinated children in studies from the south-west and eastern parts of Ethiopia were diagnosed with hepatitis B infection [7,8]. 

Variable results have been reported from other sub-Saharan Africa countries. Rey-Cuille et al. concluded that among Senegalese and Cameroonian children fully vaccinated during infancy and aged 4 years or less and born to hepatitis B-negative mothers, 58% and 92%, respectively, had demonstrated the persistence of vaccine-induced immunity [10]. Elsewhere, only 44% of Iranian children aged 6–18 years had positive antibody titers, with a notable drop in immunity after 13 years of age [11]. In a small-scale study from the U.S., only one fifth of children retained at least 10 mIU/mL of anti-HBs at six years of age, and nearly one half had no detectable antibody. The non-immune population near universally regained protective anti-HBs titers after booster vaccination [12]. Breakthrough hepatitis B infections were seen in 0.7% of a cohort of healthy seven-year-old fully vaccinated children in Taiwan, while overall, 43% were non-immune [13]. 

Though there is evidence of low vaccine-induced hepatitis B immunity in children in Ethiopia, there are very few studies outlining which ages are associated with the highest prevalence rates of lost immunity, considering the wide age range that pediatricians tend to (0–18 years). This study aimed to determine the prevalence of adequate hepatitis B vaccine immunity among children of a wider age group as compared to previous studies (1–18 years) who received three rounds of hepatitis B vaccination during infancy and delivered to hepatitis B-negative mothers in Addis Ababa, Ethiopia. 

## 2. Materials and Methods

### 2.1. Study Design

This was a retrospective cohort study. Results of paired serology tests for hepatitis B surface antibody and antigen performed for children aged 1–18 years in the period between July 2022 and June 2023 were analyzed. Obtaining consent was not required as analyzed data were anonymized.

The inclusion criteria were a child’s demonstrated completion of hepatitis B vaccinations during infancy and a negative serostatus for hepatitis B in the mother. The vaccination history was verified using a review of print-outs or electronic scans of the children’s vaccination cards. Ages beyond 18 years were excluded, as were children with a congenital or an acquired immune-deficiency disorder including those on long-term immune-suppressive treatment, due to differences in the wear-out of vaccine immunity as compared to immune-competent children. Children who had a history of chronic hepatitis B infection or past history of resolved hepatitis B infection were also excluded. Infants younger than 1 year old were excluded in order to avoid interference of post-exposure vaccinations with data analysis.

### 2.2. Study Subjects

The study population were those aged 1–18 years presenting to the out-patient clinics of the American medical center—a private health facility specializing in infectious diseases and travel medicine in Addis Ababa, Ethiopia. All children were otherwise healthy and were screened as a precautionary measure or due to a family member being infected with hepatitis B or hepatitis C virus.

### 2.3. Measurements

All samples collected during the study period were considered for inclusion. Microbiologic testing was performed following standard operating protocols (SOPs). Hepatitis B surface antigen tests (Intec Inc., Xiamen, China; sensitivity 100%, specificity 99.4%) and anti-hepatitis B surface antibody tests (Laboquick Inc., Izmir, Turkey; sensitivity 99%, specificity 98.7%) were utilized. The protocol of the study facility’s diagnostic laboratory dictates that 3 ml of blood be collected into serum separator collection tubes (SST) and processed. Adding a drop of buffer to three drops of specimen yields double bands for positive hepatitis B surface antigen test and single bands at the control level interpreted as negative results. All positive results undergo confirmatory testing with hepatitis B surface antigen ELISA test. Insertion of an anti-hepatitis B surface antibody (anti-HBsAb) test strip into a specimen will yield either a positive result (two distinct lines), indicating titers of >10 IU/mL (optimal levels) or a negative one (a single line appearing in the control region), indicating anti-HBs antibody titers of <10 IU/mL (low titers). Quantitative determination of titers was not accessible. Anti-HBc testing is performed at referral labs where 3 mL of serum is collected and analyzed.

A hepatitis B infection was diagnosed in the presence of a positive ELISA HBs antigen test, a negative anti-HBs antibody test and in the absence of a recent (less than three weeks prior to testing) administration of a hepatitis B vaccine. A recovered hepatitis B infection constituted a negative HBs antigen, positive anti-HBs and anti-HBc antibody tests. A vaccine-protected individual is one with a negative HBs antigen, a positive anti-HBs antibody test and a negative anti-HBc antibody test. An uninfected and one who is not vaccine-protected is an individual with negative HBs antigen, anti-HBs antibody and anti-HBc antibody tests. 

### 2.4. Data Analysis

All recorded data were transferred to SPSS version 29.0. The prevalence of intact hepatitis B vaccine immunity and prevalence rates in sub-groups were summarized using descriptive statistics, frequencies and tables. The magnitude of association between the different variables and vaccine-induced hepatitis B immunity was assessed using logistic regression. Statistically significant differences were taken at *p* < 0.05.

### 2.5. Ethical Considerations

The study protocol was approved by the research and publications committee of the department of pediatrics and child health of St. Paul’s Hospital Millennium Medical College, Addis Ababa, Ethiopia.

## 3. Results

A total of 269 children were considered for inclusion into the study. Following exclusion of two children with a history of chronic hepatitis B infection and a further one and ten children with inborn and acquired forms (diabetes mellitus, malnutrition, a malignant comorbidity, etc.) of immune compromise, respectively, 256 children (108 females and 148 males) were included in the study. A total of 83 children were 1–5 years old, 96 were aged 6–10 years and 77 were adolescents (11–18 years of age). Their mean age was 7.53 years (Table 1).

Six children (2.3%) had breakthrough hepatitis B infections. Three were assessed to have resolved acute hepatitis B infection following evaluation for being contacts of recently diagnosed patients and undergoing serologic and viral load measurements despite being asymptomatic. These were included in the category of children with optimal titers and underwent further univariate and multivariate analysis along with other immune uninfected children. The other three children were diagnosed with chronic hepatitis B infection following similar post-exposure assessments. These were included in the category of the study population with low titers to undergo further analysis (Table 1). The determination of the strains of infecting hepatitis B viruses was not possible. 

**Figure 1:**
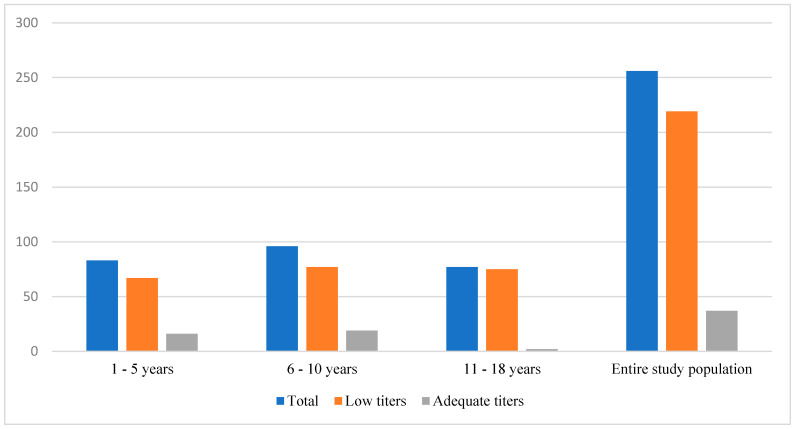
Anti-HBs antibody levels in each age group.

Overall, 37 children (14.4%) were categorized as having adequate vaccine-induced anti-HBs antibody levels (>10 IU/mL), while 219 (85.6%) had sub-optimal titers of <10 IU/mL (Figure 1). Nearly all (97.4%) of the sub-group aged 10 years and above had low seroprotection, with adolescents (11–18 years) being ten-fold more likely to have low anti-HBs antibody levels than those aged less than 5 years. Subjects with low titers were advised on receiving booster vaccinations and repeat serologies.

## 4. Discussion

The findings of this study point to a markedly high proportion (85%) of children and adolescents in Ethiopia having suboptimal anti-hepatitis B surface antibodies and thus at possible risk for breakthrough infections (which were seen in 2.3% of the study population). These figures were notably higher than those identified in studies from similar high-burden African, Asian and South American countries that we found upon performing a narrative review on the PubMed database (Table 2). 

Our study showed that adolescents aged 10 years and above were at significantly higher risk of lost seroprotection. Markedly low hepatitis B vaccine seroprotection among adolescents who completed their vaccinations during infancy was also witnessed in various other African countries according to a recently published systemic review [21]. Peak anti-HBs concentration achieved after primary vaccination and age-dependent decay in anti-HBs titers play key roles in protection against infection [16]. Breakthrough infection rates stood at 2.3% in our study population, mimicking findings from Ghana and Burkina Faso [19,20].

There are a few reasons attributed to low vaccine-induced antibodies, the loss of vaccine immunity and, in a subset of those with low titers, breakthrough infections. A large prospective cross-sectional study on 3500 children in Pakistan, which demonstrated that a third of hepatitis B-infected children occurred among fully vaccinated children born of HIV-negative mothers, postulated that improper immunization techniques or poor vaccine storage could be underlying reasons [2]. Importantly, the failure to store and handle vaccines properly on each step can reduce vaccine potency, resulting in inadequate immune responses in patients and poor protection against the disease [22]. Other reasons put forward include the differences in vaccine strain vs. infective strains (vaccine produced from an HBV genotype A2 strain not fully protective from HBV genotype non-A2 or B and C strains), emergence of S gene mutants (notably sG145R and sT126A/S mutation), immunosuppression, inadequate vaccine dosing and immediate post-vaccination infection, within the first six months of receiving the first rounds of vaccine [12,23,24,25,26]. Socioeconomic disadvantage yielded conflicting correlations with low seroprotection among studies from Egypt, Ethiopia, Italy and China [15,16,27,28]. 

As the hepatitis B vaccines in Ethiopia are administered as part of a three-dose pentavalent vaccine series, our findings call for increased scrutiny of the adequacy of vaccine-induced antibodies mounted by the remaining four components of the pentavalent vaccines: diphtheria, tetanus, pertussis (DTwP) and Haemophilus influenzae type b (Hib). The documentation of a completed vaccine schedule may not be enough in ensuring immunity against hepatitis B infections. Considering the high burden of chronic hepatitis B in Ethiopia and other low-to-middle income countries, as well as the high long-term costs associated with chronic hepatitis B and its consequences, active surveillance in high-risk patients may be considered by public health authorities.

Our study is limited by the lack of further investigations into other immunological memory pathways in immunized patients. Resolved infections are also identified in those with negative HBs antigen and anti-HBs antibody tests but positive anti-HBc antibody titers. Though protection against infection may persist beyond a drop in detectable antibodies [29], the diagnoses of breakthrough infections may indicate a real non-response to vaccination in these children, and not just a mere reduction in IgG levels. A further limitation is that testing in the study population was related to a history of exposure and risk assessment and hence is not representative for the wider pediatric-age Ethiopian population. This suggests the need for active surveillance, especially in adolescence and where indicated, the administration of booster vaccines or a complete vaccine series to prevent asymptomatic infections. 

## 5. Conclusions

In conclusion, our study highlights a potentially significant public health problem in Ethiopia. Further immunologic studies and a thorough analysis of vaccine storage and administration should be conducted to aid prevention efforts against hepatitis B infections. 

## Figures and Tables

**Table 1:** Characteristics of the study population.

Parameter (n = 252)	Anti-HBs Ab ≧ 10 IU/mL (% with Optimal Titers) (n = 37)	*p*-Value	Odds Ratio (95% CI)
Gender			
Female (n = 108)	13 (12%)	0.439	0.747 (0.357–1.563)
Male (n = 148)	24 (16.2%)		
Age groups			
1–5 years (n = 83)	16 (19.3%)	0.001	
6–10 years (n = 96)	19 (19.8%)		0.112 (0.025–0.504)
11–18 years (n = 77)	2 (2.6%)		0.108 (0.024–0.480)

**Table 2:** Comparison of rates of hepatitis B vaccine immunity and breakthrough infections.

Study	Country	Year Hep B Vaccine Introduced *	Study Year	Age Group Studied	% with Low Titers (High-Risk Groups)	% Break-Through Infections
Rey-Cuille et al. [10]	Cameroon	2005	2009–2010	<4 years	8% (higher in malnourished children)	Not determined
Gomes et al. [14]	Brazil	1997	2014–2016	3–5 years	32% (higher in older children and with longer gap time between doses)	Not determined
Zanetti et al. [15]	Italy	1991	2005	10 years	36%	Not determined
Rey-Cuille et al. [10]	Senegal	2004	2009–2010	<4 years	42% (higher in malnourished children)	Not determined
Salama et al. [16]	Egypt	1992	2010–2013	9 months–16 years	42.8% (higher in older age and females)	0.36%
Ijaz et al. [17]	Mongolia	1991	2006		46%	Not determined
Avdicova et al. [18]	Slovakia	1997	2010	11–12 years	51.6%	Not determined
Hagan et al. [19]	Ghana	2002	2012–2013	5–21 years	80.2%	2.6%
Alemayehu et al. **	Ethiopia	2005	2022–2023	1–18 years	85% (higher in adolescents)	2.3%
Barro et al. [20]	Burkina Faso	2006	2013	<6 years	94%	2.6%

* Universal vaccination for all infants; ** this study.

## Data Availability

The dataset is are not publicly available in accordance with the terms of ethical approval but are available upon request to the corresponding author.
